# Predicting the onset and persistence of episodes of depression in primary health care. The predictD-Spain study: Methodology

**DOI:** 10.1186/1471-2458-8-256

**Published:** 2008-07-25

**Authors:** Juan Ángel Bellón, Berta Moreno-Küstner, Francisco Torres-González, Carmen Montón-Franco, María Josefa GildeGómez-Barragán, Marta Sánchez-Celaya, Miguel Ángel Díaz-Barreiros, Catalina Vicens, Juan de Dios Luna, Jorge A Cervilla, Blanca Gutierrez, María Teresa Martínez-Cañavate, Bárbara Oliván-Blázquez, Ana Vázquez-Medrano, María Soledad Sánchez-Artiaga, Sebastia March, Emma Motrico, Victor Manuel Ruiz-García, Paulette Renée Brangier-Wainberg, María del Mar Muñoz-García, Irwin Nazareth, Michael King, the predictD group

**Affiliations:** 1 Departamento de Medicina Preventiva, Universidad de Málaga; Unidad de Investigación de Atención Primaria de Málaga (redIAPP, grupo SAMSERAP), Centro de Salud El Palo, Spain; 2 Facultad de Psicología. Universidad de Málaga; Fundación IMABIS; Distrito Sanitario Málaga. Unidad de Investigación de Atención Primaria de Málaga (redIAPP, grupo SAMSERAP), Spain; 3 Departamento de Psiquiatría y Medicina legal, Universidad de Granada; Grupo Andaluz de Investigación en Salud Menta, Granada, Spain; 4 Departamento de Medicina y Psiquiatría, Universidad de Zaragoza; Centro de Salud Casablanca. (redIAPP, grupo Aragón), Zaragoza, Spain; 5 Servicio Riojano de la Salud; Unidad Docente de Medicina Familiar y Comunitaria de La Rioja, Logroño, La Rioja, Spain; 6 Servicio Madrileño de Salud; Área I de Atención Primaria, Unidad Docente de Medicina Familiar y Comunitaria, Madrid, Spain; 7 Servicio Canario de Salud, Gerencia de Atención Primaria de Gran Canaria, Centro de Salud Vecindario, Las Palmas, Spain; 8 Instituto Balear de la Salud; Unidad Docente de Medicina Familiar y Comunitaria de Mallorca, Centro de Salud son Serra-La Vileta, Palma de Mallorca, Illes Balears, Spain; 9 Departamento de Bioestadística (redIAPP, grupo SAMSERAP), Universidad de Granada, Spain; 10 Unidad Docente de Medicina Familiar y Comunitaria de Granada (redIAPP, grupo SAMSERAP), Spain; 11 Instituto Aragonés de Ciencias de la Salud, Unidad de Investigación de Atención Primaria (redIAPP, grupo Aragón), Zaragoza, Spain; 12 Servicio Madrileño de Salud, Área 6 de Atención Primara, Centro de Salud Condes de Barcelona-Boadilla, Madrid, Spain; 13 Instituto Balear de la Salud, Unidad de Investigación de Atención Primaria de Baleares (redIAPP, grupo Baleares), Mallorca, Spain; 14 Servicio Andaluz de Salud, Distrito Sanitario Málaga. Unidad de Investigación de Atención Primaria de Málaga (redIAPP, grupo SAMSERAP), Spain; 15 Medical Research Council General Practice Research Framework, London, UK; 16 Department of Mental Health Sciences, UCL, London, UK

## Abstract

**Background:**

The effects of putative risk factors on the onset and/or persistence of depression remain unclear. We aim to develop comprehensive models to predict the onset and persistence of episodes of depression in primary care. Here we explain the general methodology of the predictD-Spain study and evaluate the reliability of the questionnaires used.

**Methods:**

This is a prospective cohort study. A systematic random sample of general practice attendees aged 18 to 75 has been recruited in seven Spanish provinces. Depression is being measured with the CIDI at baseline, and at 6, 12, 24 and 36 months. A set of individual, environmental, genetic, professional and organizational risk factors are to be assessed at each follow-up point. In a separate reliability study, a proportional random sample of 401 participants completed the test-retest (251 researcher-administered and 150 self-administered) between October 2005 and February 2006. We have also checked 118,398 items for data entry from a random sample of 480 patients stratified by province.

**Results:**

All items and questionnaires had good test-retest reliability for both methods of administration, except for the use of recreational drugs over the previous six months. Cronbach's alphas were good and their factorial analyses coherent for the three scales evaluated (social support from family and friends, dissatisfaction with paid work, and dissatisfaction with unpaid work). There were 191 (0.16%) data entry errors.

**Conclusion:**

The items and questionnaires were reliable and data quality control was excellent. When we eventually obtain our risk index for the onset and persistence of depression, we will be able to determine the individual risk of each patient evaluated in primary health care.

## Background

### Depression as a public health problem

In 2001 depression was the third leading cause of disease burden in high-income countries [1]. In 2004 the total annual cost of depression in Europe was estimated to be 118 billion euros, or 253 euros per inhabitant [2]. The prevalence of major depression is about 7% in the community [3] and 14% in general practice attendees [4]. Relapse is frequent up to 10 years after the first presentation [5] and residual disability is common [6].

### Risk factors for depression

The prevalence of depression is determined by exposure to risk factors that precipitate or maintain episodes of depression. With few exceptions, the prevalence and incidence of depressive disorders are higher in females than males, beginning at mid-puberty and persisting through adult life, although the determinants of gender differences are far from being established [7]. Socio-economic risk factors that might conceivably be addressed include low income and financial strain [8], unemployment [9], work stress [10], social isolation [11] and poor housing [12]. Relative poverty and unemployment are associated with a longer duration of episodes of depression rather than their onset [8], and depressive symptoms are also associated with subsequent unemployment and loss of family income [13]. Fixed factors such as a family history of depression [14] and personality [15] play a part but it is uncertain whether they act independently of other risk factors [16]. Physical health has been related to the onset and persistence of depression [17]. Stressful life events pose a greater risk for depression among women compared to men [18] and, like social support [19], these events also seem to be both a cause and a consequence of depression [20]. Many other candidate risk factors for depression exist, including for example childhood social disadvantage [21], childhood maltreatment [22], cigarette smoking [23], alcohol and drug abuse [24], and anxiety disorders [25].

However, the study of risk factors for depression suffers from limitations: First, it is often difficult to distinguish between their effects on the onset and on the course of depression; second, several risk factors may interact and be either a cause or a consequence of depression; and finally, few studies have controlled for candidate risk factors with comprehensive models, possibly due to the many possible factors involved.

### Depression risk indexes

Effective strategies for preventing depression and reducing disease burden are hindered by a dearth of evidence about whether the risk for major depression can be quantified in the same way as other clinical disorders, such as cardiovascular diseases [26].

The predictD study is a pioneering international study whose main objective was to develop a risk index for the onset of episodes of major depression in general practice attendees [26]. The predictD international study recruited and followed-up a large sample of general practice attendees over one year. From 39 potential risk factors for depression, a risk index of 10 risk factors was drawn up with an excellent predictive power and good external validity [27].

### The predictD-Spain study

Drawing on our experience as part of the predictD international study [28], the predictD-Spain study aimed to improve certain methodological aspects, extending the follow-up for three years, considering genetic factors in the equation (the predictD-Gene study), and studying professional and organizational factors as contributors to both the onset and persistence of episodes of depression (the predictD-Services study).

A genetic predisposition to depression may be a potential risk factor in the development of depression. Although the neurobiological equivalent of the predisposition remains unclear, the brain's serotonin system appears to play an important mediating role. Individuals with the 5-HTTLPR s/s genotype are more prone to develop depression [29], and this genotype may determine stress coping mechanisms and thereby increase stress vulnerability [30].

A recent systematic review identified only 17 longitudinal studies of depression in primary care, most of which involved small sample sizes or were relatively short-term [31]. The most usual risk factors for persistence of depression in primary care were severity and chronicity of the depressive episode, the presence of suicidal thoughts, poorer self-reported quality of life, lower self-reported social support, experiencing key life events, antidepressant use, lower education level and unemployment. However, whether differences exist in depression outcomes between patients whose depressive disorders are recognized and those whose disorders are unrecognised in primary health care is unclear [32].

In Spain, general practitioneers (GPs) failed to detect 30% of depressed patients [33], while only 30% of those diagnosed received appropiate treatment [34]. The suitable use of antidepressants, medication adherence, and 'case management' between mental health specialists and primary care professionals might be some of the best predictors for the recovery from depression [35]. Thus, the need to control for these factors in predictive models on the persistence of depression in primary care is clear [36].

Each anxiety disorder and panic attack appears to confer an independent risk for the onset of major depression [25] and an association between psychopharmacological treatment for generalized anxiety disorders and a lower risk of depression has been suggested [37]. However, anxiety disorders are not always adequately detected and managed by GPs [38]. Consequently, the detection and treatment of anxiety disorders might also condition the onset of depression.

Several GP factors are related with their ability to detect and manage psychosocial problems: gender, interview training, previous doctor-patient relationship or psychosocial orientation [39-42]. Concerning organizational factors, a recent meta-analysis showed that collaborative care for depression improved the outcome [43]. The most commonly used intervention features in the collaborative care were patient education and self-management, monitoring of depressive symptoms and treatment adherence, decision support for medication management, a patient registry, and mental health supervision of care managers [44]. Finally, one of the best professional-organizational factors associated with the recognition and good management of psychosocial problems in primary care is the length of interviews [42,45].

Accordingly, we aim to develop comprehensive models to predict the onset and persistence of episodes of depression in primary care. As well as individual, genetic, and environtmental risk factors, we are also considering other professional and organizational factors. In this report we explain the general methodology of the study and evaluate the reliability of the questionnaires used.

## Methods/Design

### Design

This prospective cohort study has recruited a systematic random sample of general practice attendees to be followed up after 6, 12, 24 and 36 months. The prevalence of depression and risk factors for depression are to be assessed at baseline and at each follow-up point. After excluding the patients with depression at baseline, the incidence of depression is to be measured at 6, 12, 24 and 36 months. This project is in compliance with the Helsinki Declaration and the relevant ethics committees in each province have approved the study. In representation of them, from the coordinator centre, the "*Comité de Ética e Investigación Sanitaria del Distrito Sanitario de Atención Primaria de Málaga*" approved the study.

### Setting

Seven provinces are participating with 41 health centres and 231 GPs distributed throughout Spain: Malaga and Granada in southern Spain; Zaragoza and La Rioja in northern Spain; Madrid, capital of Spain, situated in the centre; Las Palmas in the Canary Islands; and Majorca in the Balearic Islands. Each health centre, which covers a population of 15,000 to 30,000 inhabitants from a geographically defined area, is staffed by GPs, who see patients over the age of 14 years, and by primary care paediatricians. The GPs in each health centre work as a group, with extensive primary care teams. The Spanish National Health Service provides free medical cover to 100% of the population. The health centres taking part extend over urban and rural settings in each province.

### Sample and exclusion criteria

A systematic random sample taken at regular intervals of between 4 and 6 attendees, aged 18 to 75, has been recruited in six Spanish provinces. The 7^th ^province, Malaga, started between October 2003 and February 2004 because it was already participating in the predictD international study [26]. The GPs introduce the study to the selected patients and request permission before contacting the assistant researcher. Patients over 75 years of age have been excluded because the prevalence of cognitive impairment increases after that age. Other exclusion criteria include inability to speak or understand Spanish (foreigners), severe organic mental disease and terminal illness, patients due to be away for more than 3 months during the coming year, and persons (representatives) who attend the surgery on behalf of the person who has the appointment (for example, to collect a prescription or a certificate). Participants who have given informed consent have an interview at the health centre within two weeks. The sample size was computed, using Obuchowski expression [46], to estimate the area under the ROC curve of the index to be obtained. Assuming an area under the ROC curve of 0.80 and a precision of 0.06, and considering an intraclass correlation coefficient of 0.05 with an average of 11 patients per GP in the cluster, the sample size needed was 3,474 patients for an incidence of major depression of about 12% per year.

### Outcome measures

Our outcome variable is a depressive disorder. Depression is measured with the 12-month (or modified to 6-month) Depression Section of the Composite International Diagnostic Interview (CIDI) [47-49], which provides psychiatric diagnoses according to ICD10 and DSM-IV.

### Risk factors for depression

The selection of risk factors for the onset and persistence of depression was designed to cover all important areas identified in a systematic review of the literature, considering specially those assessed in the predictD international study [26], in addition to other possible professional and organizational risk factors. Where possible, we used published measures with established reliability and international validity, including in Spain. Where this was not possible we translated the measures into Spanish. Each translation was back-translated by professional translators. In some cases, questions were developed for the study or adapted from available standardised instruments. These questions were evaluated for test-retest reliability. Scales without validation data in Spain were also evaluated on their internal consistency and factorial validity.

#### Individual and environmental risk factors

• Socio-demographic factors: age, marital status, occupation, employment status, ethnicity, nationality, country of birth, educational level, income, owner occupier accommodation, living alone or with others.

• Controls, demands and rewards for unpaid and paid work, using an adapted version of the job content instrument [50].

• Debt and financial strain [9].

• Consultation rate in the general practice through computerized clinical notes [51].

• Physical and mental well-being, assessed by the SF-12 that has application across a number of cultures [52], including Spain [53]; and a question on the presence of long-standing illness, disability or infirmity.

• Alcohol abuse using the WHO AUDIT questionnaire [54], the Spanish validation of which slightly modified the threshold for female hazardous drinkers [55,56].

• Use of recreational drugs (at least once in the past and over the previous six months) adapted from the relevant sections of the CIDI.

• A life-time screen for depression based on the first two questions of the CIDI. People answering yes to both questions screened positive [57].

• Brief questions on cigarette consumption.

• For women, questions on menstruation, pregnancy and childbirth from the Patient Health Questionnaire (PHQ) [58].

• Brief questions on the quality of sexual and emotional relationships with a partner, adapted from a standardized questionnaire [59].

• Presence of serious physical, psychological or substance misuse problems, or any serious disability, in persons who are close friends or relations of participants; and difficulty getting on with people and maintaining close relationships, assessed using questions from a social functioning scale [60].

• Childhood experiences of physical, emotional or sexual abuse [61].

• Nature and strength of spiritual beliefs [62].

• Family psychiatric history in first-degree family members requiring pharmacological or psychological treatment in primary or secondary care, and suicide in first-degree relatives [63].

• Anxiety symptoms using the anxiety section of the PRIME-MD [58]. The Spanish version provides psychiatric diagnoses according to DSM-IV: Panic Attack, Generalised Anxiety Disorder and Other Anxiety Disorders [64].

• One question on whether and when (at what age) the participant had lost one or both parents by death.

• Household type and composition.

• The living environment, including satisfaction with neighbourhood and perception of safety inside/outside the home using questions from the Health Surveys for England [65].

• Recent life-threatening events, using a brief validated checklist [66].

• Experience of discrimination on the grounds of sex, age, ethnicity, appearance, disability or sexual orientation using questions from a recent European study [67].

• Adequacy, availability and sources of social support from family and friends [68].

#### Genetic risk factors

The participants give their general informed consent and are asked for a new and specific informed consent on genetic tests. We collect saliva and/or blood for genetic testing, with DNA from both blood and saliva obtained by standard procedures. The 5-HTTLPR polymorphism at SLC6A4 is to be genotyped in all samples, as described [29,30].

#### Professional and organizational risk factors

This group of variables will be gathered from computerised clinical notes, centralised administrative records, and a brief questionnaire to the GPs at 12, 24 and 36 months.

• GP characteristics: age, gender, year of degree in Medicine, postgraduate training and speciality, type of contract, time in the current health centre, list size, mean time per patient during the previous year, satisfaction with relationships and collaborative care between GP and mental health team, social worker, and nurse practitioner, self-perceived comfort with antidepressant use, and a questionnaire on professional satisfaction, perception of workload, and psychosocial orientation [69].

• Health Centre characteristics: size of population attended, number of inhabitants in the city or town, predominant activity in the city or town (agriculture-fishing, industry or services), number and type of professionals in the team, professional-population ratios, type and intensity of relationship with Mental Health team (case management, patient care and shared continued medical education), and "centred variables" (mean or median of the GP characteristics in each health centre).

• Interaction professional-organization-patient variables: number of visits to health centre team, i.e., GP, nurse, and social worker; referrals to the Mental Health team by GPs or direct approaches to mental health specialists by the patient privately; patient's psychosocial and physical problems detected by their GPs; and antidepressants, benzodiazepines or other psychological drugs prescribed (type, dose and duration).

### Data checking

Locally, each interview is checked for completion by the interviewer. Quality assurance is focused on the standardised training of researchers in the use of the CIDI and other questionnaires, on the recruitment and interviewing of patients and on data management. Over and above team meetings at the provincial level, a research coordinator assesses each interviewer twice during recruitment to monitor the interview process, verifies adherence to the CIDI and manages other problems as they arise. Progress reports for each province are submitted every two months and examined critically by the steering group at project management meetings. Each participating province double-enters 10% of its data records and a 1% error rate is accepted.

### Statistical analysis

When all follow-ups are complete we shall be able to identify risk factors for the incidence (from participants not depressed at baseline) and recovery-persistence (from depressed participants) of depression over 6, 12, 24 and 36 months. The occurrence of major depression (yes/no) will be the dependent variable of multiple logistic regressions from a multilevel analysis that will discern three levels: patient, GP and health centre. The province will be included as a fixed factor since only seven units are involved. When we consider repeated measurements from different times of the follow-up, multilevel analysis will include four levels: time, patient, GP and health centre. A hierarchical model will be used to take into account the distribution of the data at different levels to estimate two types of variability, one due to individuals in the study and another due to the groups in which patients are nested. The candidate risk factors in each level will be included in the model using an entrance value of *P *< 0.10. For each level, the usefulness of including first-degree interactions in the equation will be considered, and the interactions between levels will also be studied.

Missing data for candidate risk factors will be imputed using multiple imputation by chained equations (MICE), in which each variable is imputed using a regression model conditional on all the others, iteratively cycling through all the variables that contain missing data [70]. We will conduct the analysis in each of 10 imputed datasets and will obtain combined estimates; 10 imputed data sets is a common choice by convention [71]. We will repeat the analysis in just those participants with complete information as a sensitivity analysis. These analyses will be performed by the STATA "ice" command [72].

We will calculate the c-index [73] to estimate the discriminative power of the final model at each time. We will use a calculation proposed by Copas [74] to adjust for over-fitting of our prediction models. We will assess the goodness of fit of the final risk model by grouping individuals into deciles of risk and comparing the observed probability of major depression within these groups with the average risk. We will calculate effect sizes using Hedge's g [75] for the difference in log odds of predicted probability between patients who will later be observed to be depressed and those who will not be depressed. All these analyses will be run with STATA release 10.

We have calculated test-retest agreement using the Kappa statistic, which adjusts for chance agreement, for questions with two response options and the Intraclass Correlation Coefficient (ICC) for items with more than two [76]. We have evaluated the internal consistency of the scales through Cronbach's alpha, and explored their subjacent factors through factorial analysis by principal components and varimax rotation. Reliability and validity analyses have been run with SPSS 14.

## Results

### Reliability

For the test-retest analysis, we selected a random sample of 401 patients stratified by province; 251 completed the predictD-Spain questionnaires as researcher-administered and 150 as self-administered questionnaires between October 2005 and February 2006. The respective distribution by province was: Madrid 41/39, Granada 40/38, La Rioja 49/24, Las Palmas 42/20, Malaga 41/0, Majorca 28/19, and Zaragoza 10/10. The mean number of days between test and retest was 11.0 (95% CI, 10.2–11.8; standard deviation = 7.5).

Additional file 1 shows reliability coefficients for all items and questionnaires evaluated. Most of the coefficients were good or excellent. Use of recreational drugs over the previous six months had poor agreement for the two methods of questionnaire administration. One item on the perception of safety inside the home had a coefficient of 0.80 (excellent) for researcher-administered and 0.37 (poor) for self-administered questionnaires, and another on cigarette consumption also had poor agreement (researcher-administered = 0.85 and self-administered = 0.40), although only for the self-administered way.

We evaluated the internal consistency of three scales: dissatisfaction with unpaid work, dissatisfaction with paid work and social support from family and friends. The respective Cronbach's alphas were good (Additional file 1).

### Validity

Factorial analysis of the scale dealing with social support from family and friends found one factor that explained 58.7% of the variance. Factorial analysis of the scale dealing with dissatisfaction with paid work found three factors that explained 81.6% of the variance: F1 "feeling in control" (3 items = 28.3%), F2 "experiencing difficulty without support" (2 items = 26.4%), and F3 "experiencing distress without being respected" (2 items = 26.9%). Factorial analysis of the scale dealing with dissatisfaction with unpaid work differed slightly from the scale dealing with paid work. It found two factors that explained 77.3% of the variance: F1 "feeling in control without difficulties and with gratitude" (5 items = 50.4%) and F2 "experiencing distress without support and being respected" (2 items = 26.9%).

### Error rates for data entry

The baseline error rates for data entry in each province are well below the 1% level of acceptability. We have checked 118,398 items from a random sample of 480 patients stratified by province and found only 191 errors (0.16%).

## Discussion

The questionnaires used showed good reliability and the factorial validity of the three scales tested was coherent. Quality control of data was excellent.

### Reliability and validity analysis

The results of reliability analyses were good or excellent for practically all the questionnaires and items. This suggests that data stability over time is satisfactory. We were interested in testing the questionnaires using both methods of administration, researcher-administered and self-administered, since, for cultural reasons; questionnaires administered to primary care attendees in Spain are almost always researcher-administered. Moreover, we also wanted to test whether self-administered questionnaires have different degrees of reliability as a result of a different socio-cultural patient profile (higher level of education, income and social class), the variability introduced by interviewers, or other circumstances. In the end, the test-retest reliability was similar for most items with both methods of administration, although we did not measure inter-interviewer reliability. Nevertheless, several differences were interesting. The perception of safety inside the home had an excellent coefficient for researcher-administered questionnaires and a poor coefficient for self-administered questionnaires. Perhaps not all the patients interpreted this question the same way or maybe some were less sincere. We think that it was probably a "hot" question, perhaps interpreted as domestic violence, and, when the interviewers were not directly present; the patient may have felt less need to be sincere. It might also be that the question was a bit ambiguous. Fewer doubts concerned the absence of reliability for the item on the use of any recreational drugs over the previous 6 months. It contrasted with the good reliability of the same question in reference to the use of recreational drugs at least once. From the viewpoint of social desirability it is coherent, as it is considered better to try a drug than to consume it more often. A similar reasoning could be attributed to the item on cigarette consumption per day, although it is only a hypothesis. However, the predictD-International study obtained good coefficients for both time references on drug consumption [26]. In any case, we have decided not to include the use of recreational drugs over the previous six months in the final analysis of risk factors. The items on perception of safety inside the home and consumption of cigarettes per day will also be excluded from the final analyses.

Factorial analysis and Cronbach's alpha of the scale dealing with support from family and friends showed that its use as a single scale is appropriate. This is a conceptual difference with those questionnaires that separate support from family and support from friends [77]. The 7 items that we used on dissatisfaction with paid and dissatisfaction with unpaid work were adapted from the "Job Content Questionnaire" (JCQ), based on Karasek's demand-control-support model [78]. Our factorial analysis of paid work identified three factors that coincide with those obtained by the JCQ in different countries (job control, social support and psychological demands). However, factorial analysis of unpaid work changed. This was to be expected, as most unpaid work was probably related with looking after the family or the home, though we are unaware of any background to this question in the literature. Whatever the case, both Cronbach's alphas justified the use of each scale as a single scale.

### Significance and practical implications of the study

These comprehensive models for the prediction of depression will facilitate the understanding of its causes, specifying the contribution of each risk factor and the importance of patient, environmental, genetic, professional and organizational factors. When we eventually obtain our risk index for the onset of depression, we will be able to determine the individual risk of each patient evaluated in primary health care. We hope to produce a simple prediction tool similar to the cardiovascular risk index [79]. Additionally, we plan to develop another risk index for recovery from depression, thus laying the foundation for future research on risk reduction in primary care.

## Abbreviations

GPs: General Practitioners; ICD10: International Classification of Diseases, version 10; DSM-IV: Diagnostic and Statistical Manual of Mental Disorders, 4^th ^edition; WHO: World Health Organization; AUDIT: Alcohol Use Disorders Identification Test; PRIME-MD: Primary Care Evaluation of Mental Disorders.

## Competing interests

The authors declare that they have no competing interests.

## Authors' contributions

JAB is guarantor for the predictD-Spain study. JAB, BM-K, FT-G, and CM-F obtained funding for implementing the study in Spain. The predictD-Spain study was designed and based on an idea by MK, IN, and the predictD-Europe core group. JAB coordinated the predictD-Spain study. BM-K, FT-G, CM-F, MJGdG-B, MS-C, MAD-B, and CV coordinated the study in each Spanish province. JAC and BG, based on the predictD-Spain and predictD-International study, designed the sub-study predictD-Gene and obtained funding for it. MTM-C, BO-B, AV-M, MSS-A, SM-LL, EM-M, VMR-G, PRB-W collaborated implementing the study in each province. JDL collaborated in the design, and JDL and JAB analysed the data. JAB drafted the paper and all authors agreed the final version.

## Pre-publication history

The pre-publication history for this paper can be accessed here:



## Supplementary Material

Additional file 1 Table 1. Reliability analysis by predictD – Questionnaires. Click here for file
