# Supplementary material for: Predicting the onset and persistence of episodes of depression in primary health care. The predictD-Spain study: Methodology
Source: BMC Public Health. 2008 Jul 25;8:256. doi: 10.1186/1471-2458-8-256 (PMC2527330; doi:10.1186/1471-2458-8-256)
Supplement: Additional file 1 — Table 1. Reliability analysis by predictD – Questionnaires. [file 1471-2458-8-256-S1.doc]

**TABLE 1**: Reliability analysis by predictD - Questionnaires.

| **QUESTIONNAIRE** | **Number of items** | **Analysis** | **Reliability range of items**  **(researcher-administered); N=251** | | **Reliability range of items**  **(self-administered); N=150** | | **Cronbach’s Alpha @** |
| --- | --- | --- | --- | --- | --- | --- | --- |
|  |  |  | **Minimum** | **Maximum** | **Minimum** | **Maximum** |  |
| **Social-demographic variables** | 12 | Kappa & ICC | 0.85 1.00 | | 0.72 1.00 | |  |
| **Sharing current household** | 12 | Kappa | 0.82 1.00 | | 0.80 1.00 | |  |
| **Income and financial difficulties** | 4 | ICC | 0.80 0.94 | | 0.80 0.88 | |  |
| **long-standing illness, disability or infirmity** | 1 | Kappa | 0.70 | | 0.75 | |  |
| **Satisfied with living together at home** | 1 | ICC | 0.85 | | 0.83 | |  |
| **Dissatisfaction with unpaid work** | 7 | Kappa & ICC **^1^** | 0.64 0.82 | | 0.76 0.83 | | 0.73 |
| **Dissatisfaction with paid work** | 7 | Kappa & ICC **^2^** | 0.76 0.91 | | 0.74 0.91 | | 0.89 |
| **Satisfaction with neighbourhood** | 1 | ICC | 0.87 | | 0.82 | |  |
| **Perception of safety inside the home** | 1 | ICC | 0.80 | | 0.37 | |  |
| **Perception of safety outside the home** | 1 | ICC | 0.85 | | 0.86 | |  |
| **List of threatening experiences** | 12 | Kappa | 0.60 0.72 | | 0.63 0.89 | |  |
| **Social support from family and friends** | 7 | ICC **^3^** | 0.70 0.88 | | 0.84 0.90 | | 0.88 |
| **Family history of psychological disorders** | 14 | Kappa & ICC | 0.82 1.00 | | 0.78 1.00 | |  |
| **Discrimination** | 7 | Kappa | 0.50 0.72 | | 0.66 1.00 | |  |
| **Serious problems in very close persons** | 4 | Kappa | 0.63 0.72 | | 0.61 0.82 | |  |
| **Satisfaction with sexual-emotional relationships** | 3 | ICC | 0.83 0.90 | | 0.80 0.92 | |  |
| **Relationships with others** | 2 | ICC | 0.90 0.92 | | 0.76 0.83 | |  |
| **Religious and Spiritual beliefs** | 3 | ICC | 0.91 0.95 | | 0.89 0.95 | |  |
| **Childhood abuse** | 3 | ICC | 0.89 0.96 | | 0.89 0.92 | |  |
| **Recreational drug use (at least once in the past)** | 10 | Kappa | 0.54 1.00 | | 0.67 0.85 | |  |
| **Recreational drug use (previous six months)** | 10 | Kappa | 0.05 0.10 | | - 0.02 0.15 | |  |
| **Tobacco consumption questions** | 6 | Kappa & ICC | 0.86 0.99 | | 0.89 * 1.00 | |  |

*ICC: Intraclass Correlation Coefficient;* **^1^** *Scale ICC researcher-administered = 0.83; and self-administered = 0.85;* **^2^***Scale ICC researcher-administered = 0.93; and self-administered = 0.92;* **^3^** *Scale ICC researcher-administered = 0.89; and self-administered = 0.93.* ******* *One item with ICC=0.40 as self-administered was removed.* ***@*** *Only variables used as scales are shown.*
